# Enhancing Research Through Image Analysis Workshops: Experiences and Best Practices

**DOI:** 10.1002/jemt.24769

**Published:** 2024-12-17

**Authors:** Stefania Marcotti, Martin L. Jones, Thomas J. A. Slater, David J. Barry

**Affiliations:** ^1^ Randall Centre for Cell and Molecular Biophysics King's College London London UK; ^2^ Electron Microscopy Science Technology Platform The Francis Crick Institute London UK; ^3^ Cardiff Catalysis Institute, School of Chemistry Cardiff University Cardiff UK; ^4^ Advanced Light Microscopy Science Technology Platform The Francis Crick Institute London UK

**Keywords:** image analysis, open‐source software, training, workshops

## Abstract

Modern microscopy systems allow researchers to generate large volumes of image data with relative ease. However, the challenge of analyzing these data effectively is often hindered by a lack of computational skills. This bottleneck negatively impacts both research reproducibility and efficiency, as researchers frequently rely on manual or semi‐automated analysis methods. Interactive image analysis workshops offer a valuable solution, equipping researchers with the skills and tools needed to automate image processing tasks. In this paper, we share our experiences and best practices from conducting such workshops, which emphasize the use of open‐source software like ImageJ, FIJI, and Python‐based tools such as JupyterLab and napari. We discuss key considerations for workshop design, logistics, and outcomes, while highlighting common pitfalls to avoid. Using two recent workshops as case studies, we also present strategies for optimizing participant engagement and learning. Our insights offer practical guidance for planning and conducting image analysis workshops and serve as a starting point for researchers looking to establish similar training initiatives and enrich their local imaging communities.

## Graphical abstract

This graphical abstract illustrates the structure of interactive image analysis workshops, where participants engage in hands‐on learning with open‐source tools under expert guidance to improve research reproducibility.

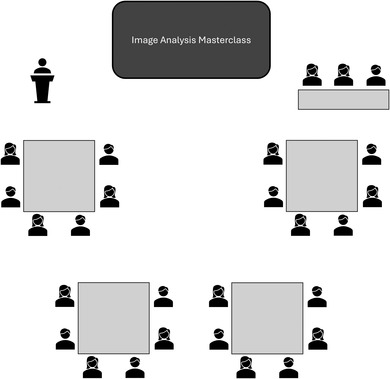

**Summary**

- Interactive workshops improve image analysis skills and enhance research reproducibility.
- We offer practical insights for designing effective workshops using open‐source tools, boosting participant engagement and avoiding common pitfalls.

## 1 Introduction

Modern microscopy systems enable researchers to routinely produce vast quantities of image data with relative ease. However, the ease of data production contrasts sharply with the challenges associated with its analysis. These challenges are further compounded by the lack of computational skills among many researchers, particularly those needed for developing robust image analysis workflows. Consequently, image analysis often becomes a bottleneck in the research process, with many researchers resorting to qualitative, manual image analysis. Such approaches have serious implications for reproducibility, are tedious and time‐consuming, and cannot be scaled effectively.

**FIGURE 1:**
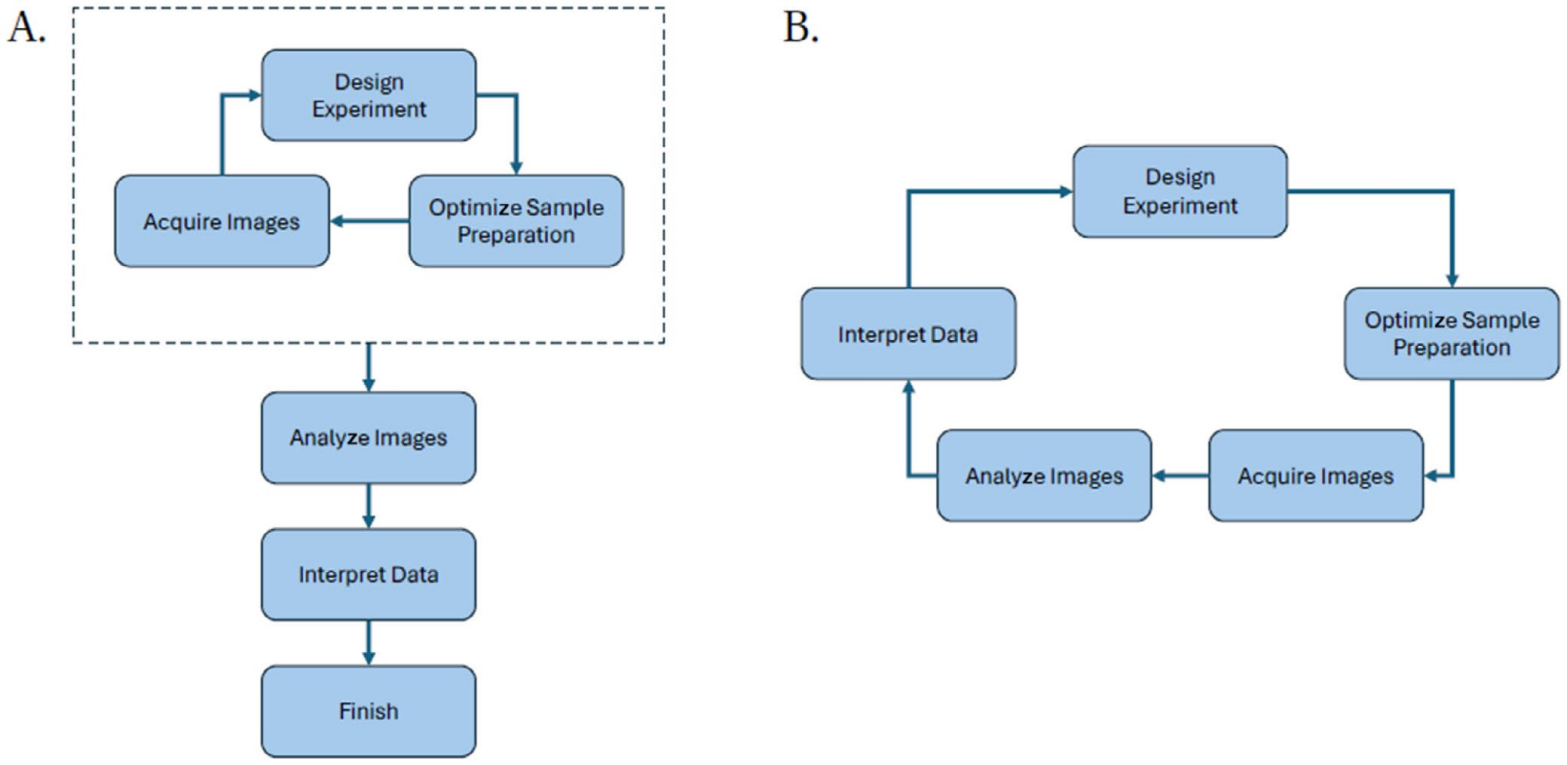
A primary goal of an image analysis workshop should be to change researchers' mindset. (A) How many researchers view image analysis—something to be considered once image acquisition has been optimized and quantitative data are required. (B) How researchers should think about image analysis—as a fundamental component of the research process (Senft et al. 2023).

Interactive workshops offer a solution to these challenges by equipping researchers with the tools needed to explore image analysis (Figure 1). By introducing researchers to popular, open‐source tools (see Box 1), these workshops empower them to automate their analyses, thereby saving significant time and ensuring consistency, accuracy, and, most importantly, reproducibility.

BOX 1 **Examples of free, open‐source software for image analysis. Which you choose to use during your workshop will depend on the aims and your target audience.**

**Table:** 

**ImageJ** (Schneider, Rasband, and Eliceiri 2012) Public domain software for processing and analyzing scientific images. https://imagej.net/ij	**CellProfiler** (Stirling et al. 2021) Software for measuring and analyzing cell images. https://cellprofiler.org
**FIJI** (Schindelin et al. 2012) A “batteries‐included” distribution of ImageJ, bundling many plugins that facilitate scientific image analysis. https://fiji.sc	**QuPath** (Bankhead et al. 2017) Offers a powerful set of tools for working with whole slide images. https://qupath.github.io
**JupyterLab** (Kluyver et al. 2016) A web‐based interactive development environment for notebooks, code, and data. https://jupyter.org	**ilastik** (Berg et al. 2019) A machine learning‐based tool for interactive image classification, segmentation, and analysis. https://www.ilastik.org
**napari** (Ahlers et al. 2023) A Python library for *n*‐dimensional image visualization, annotation, and analysis. https://napari.org	**Icy** (Chaumont et al. 2012) Provides resources to visualize, annotate, and quantify bioimaging data. https://icy.bioimageanalysis.org

Drawing from our experience of running image analysis workshops over several years, this perspective shares insights into what has worked well and what has been less successful. We provide general guidelines on planning an image analysis workshop (see Box 2) and discuss specific examples of two workshops we have recently conducted.

BOX 2 **There are many things to consider when planning an image analysis workshop for the first time.**

**Table:** 

**Who is your target audience?** PhD students? Core facility staff? Group leaders? All of the above?	**How many participants and instructors?** Aim for an instructor:participant ratio between 1:5 and 1:10 (depending on content).
**What are the prerequisites?** Are you targeting beginners? People with basic knowledge? Seasoned image analysts?	**How much will the workshop cost?** Be realistic about costs and how you intend to cover them.
**What is the aim of the workshop?** What specific task(s) should participants be able to complete?	**What kind of room will you use?** The room layout will influence the workshop format—group work is harder in a lecture theater.
**How long will the workshop be?** Longer workshops allow you to cover more material, but it might be harder to attract applicants.	**What software will participants be using?** Best to stick with what you know—teaching with unfamiliar software can be challenging, especially if things go wrong.
**What material do you want to cover?** Be specific and produce realistic time estimates.	**Will participants be bringing their own laptops?** Allows participants to easily use the same tools after the workshop, but more time will likely be needed to deal with technical issues.
**Can participants bring their own data?** May make the workshop more appealing but can significantly heighten expectations and increase workload for instructors.

## 2 Before the Workshop

### 2.1 Plan Well in Advance

When organizing an image analysis workshop, it is crucial to plan well in advance. Start by mapping out a comprehensive timeline (Table 1). Much of what follows in this manuscript assumes the workshop being planned aims to attract participants from beyond your host institution. But, as alluded to in Table 1, this may not necessarily be the case—your aim may be to host more regular workshops exclusively for students or staff at your host institute. While some specific issues raised in this article may not necessarily be relevant in this case, most of the guidance that follows is very general and will likely be of benefit to anyone planning a workshop for the first time.

**TABLE 1:** Outline of timeline for in‐person workshop with local or international participants.

Task	Suggested deadline (weeks before workshop)	
	International	Local
Identify a venue	40–50	
Confirm the date based on venue availability	40–50	
Apply for funding	40+	
Draft application form	30–40	
Set up a web page	30–35	
Finalize budget	30–35	
Publish program outline	25–30	12–26
Publicize your workshop	25–30	12–26
Schedule a test run	25–30	8–26
Open for applications	20–25	12–26
Plan social event	20–25	8–12
Make travel plans	20–25	N/A
Application deadline	15–20	8–12
Finalize program	15–20	4–8
Notify participants	15–20	4–8
Publish content	10–15	1+
Registration fee deadline	10–15	1+
Book catering	5–10	1+
Send instructions to participants	2–5	1+
Request feedback	0	0
Debrief	0–1 (after workshop)	0–1 (after workshop)
Follow up with participants	20–30 (after workshop)	4–8 (after workshop)

*Note:* Deadlines for local workshops are obviously more dependent on local institutional requirements. Additionally, some of these tasks may not necessarily be relevant, particularly for local workshops.

Allow ample time to secure a suitable venue, apply for funding (if necessary), and notify participants well in advance. This lead time ensures participants can make travel arrangements and obtain any required visas (if necessary). Expect setbacks and unexpected delays during the planning process, so the more time you allow, the better. The planning timeline will be significantly longer if your workshop involves external participants compared to an internal‐only event.

### 2.2 Who Is Your Target Audience?

Deciding on your target audience at the outset is crucial, as it will significantly influence the content of your course. For instance, are you aiming to teach complete novices the basics of image analysis? Or is your goal to target experienced users and focus on automating tasks? Clearly defining your audience helps tailor the workshop to meet their specific needs and ensures a more effective learning experience. Much of what follows in this manuscript assumes an introductory‐level workshop targeted at relative novices and this should be borne in mind throughout. Our recommendations would likely require adaptation for more advanced workshops. However, providing a different set of recommendations for each of the broad range of possible scenarios that could fall under the heading “advanced workshop” is beyond the scope of this manuscript.

In addition to the above, it is important to consider how you expect your participants to use what they learn. Are you trying to equip researchers with the tools they need to effectively conduct their research? Or do you wish to “train the trainers,” where the aim is to target, for example, core facility staff, who will in turn train researchers in their host institutions? Which you choose will likely influence the content of your course, with future trainers likely to be more interested in a broad, general introduction to the subject, while researchers will more likely be interested in finding solutions to their specific problems.

### 2.3 What Format Will You Use?

Once you have identified your target audience, you can decide on the format of your workshop. Key considerations include the duration of the workshop, the number of instructors, and the number of participants. In our experience, an instructor‐to‐participant ratio of approximately 1:5 is optimal. A lower ratio of 1:10 can also work, although it may become challenging if many participants require significant support.

Dividing participants into groups can help manage these challenges. If your audience includes both novices and experienced users, creating groups with a mix of abilities allows for peer‐to‐peer assistance, which can reduce the burden on instructors and enhance the learning experience (Laal and Ghodsi 2012). While some people can be resistant to the active learning nature of group‐based workshops, they invariably end up learning more—although the greater cognitive effort required can lead them to believe they are learning less than they would in a passive lecture (Deslauriers et al. 2019).

### 2.4 How Long Will Your Workshop Be?

This is a key consideration and will likely be influenced by the amount of time that trainers can commit, as well as cost considerations if, for example, venue hire is required, catering is being provided and participants are required to pay for accommodation. Depending on the target audience and participant expectations, participants may be limited in how much time they can spend away from their day‐to‐day activities. Conversely, a workshop may be perceived as being low value if the duration is too short and you may struggle to attract interest. We find a duration of 2–3 days for an introductory workshop to be well received by participants when compiling feedback.

### 2.5 In‐Person, Online, or Hybrid?

Whenever possible, we recommend an in‐person format for your workshop. Although virtual workshop formats have become increasingly prevalent for various reasons, our extensive experience delivering these workshops has consistently shown that in‐person formats lead to superior learning outcomes. An in‐person setting promotes spontaneous questions and real‐time feedback, fosters an environment of active learning, and provides an opportunity for participants to network with their peers.

As mentioned above, organizing participants into groups facilitates deeper discussions and collaborative problem‐solving. Such interactions are significantly more effective and organic when participants share a physical space, allowing them to engage in lively discussions around the same table. Pedagogically, it has been shown that small group work increases engagement, knowledge retention, self‐directed learning, and teamwork ability (Collaborative Learning n.d.).

Technical challenges are inevitable in workshops that rely on computers and software. Addressing these issues remotely can often be more complex and time‐consuming. In contrast, in‐person troubleshooting allows for immediate resolutions, ensuring minimal disruptions and maintaining the workshop's momentum.

However, it is essential to consider the broader research community's interest and take steps to ensure accessibility. Offering a hybrid format can help to provide accessibility to those with caring responsibilities, health restrictions, or who cannot travel for other reasons. While in‐person settings offer unique benefits, committed individuals can still achieve significant learning outcomes through online courses and videos, even if the learning curve may be steeper. At a minimum, make your training materials available online. Recording some aspects of the workshop and publishing them online can also be beneficial, although this may require substantial video editing before publication. This approach ensures that the workshop remains inclusive and accessible to all participants, regardless of their circumstances.

### 2.6 Be Sure the Training Room Is Appropriate

**FIGURE 2:**
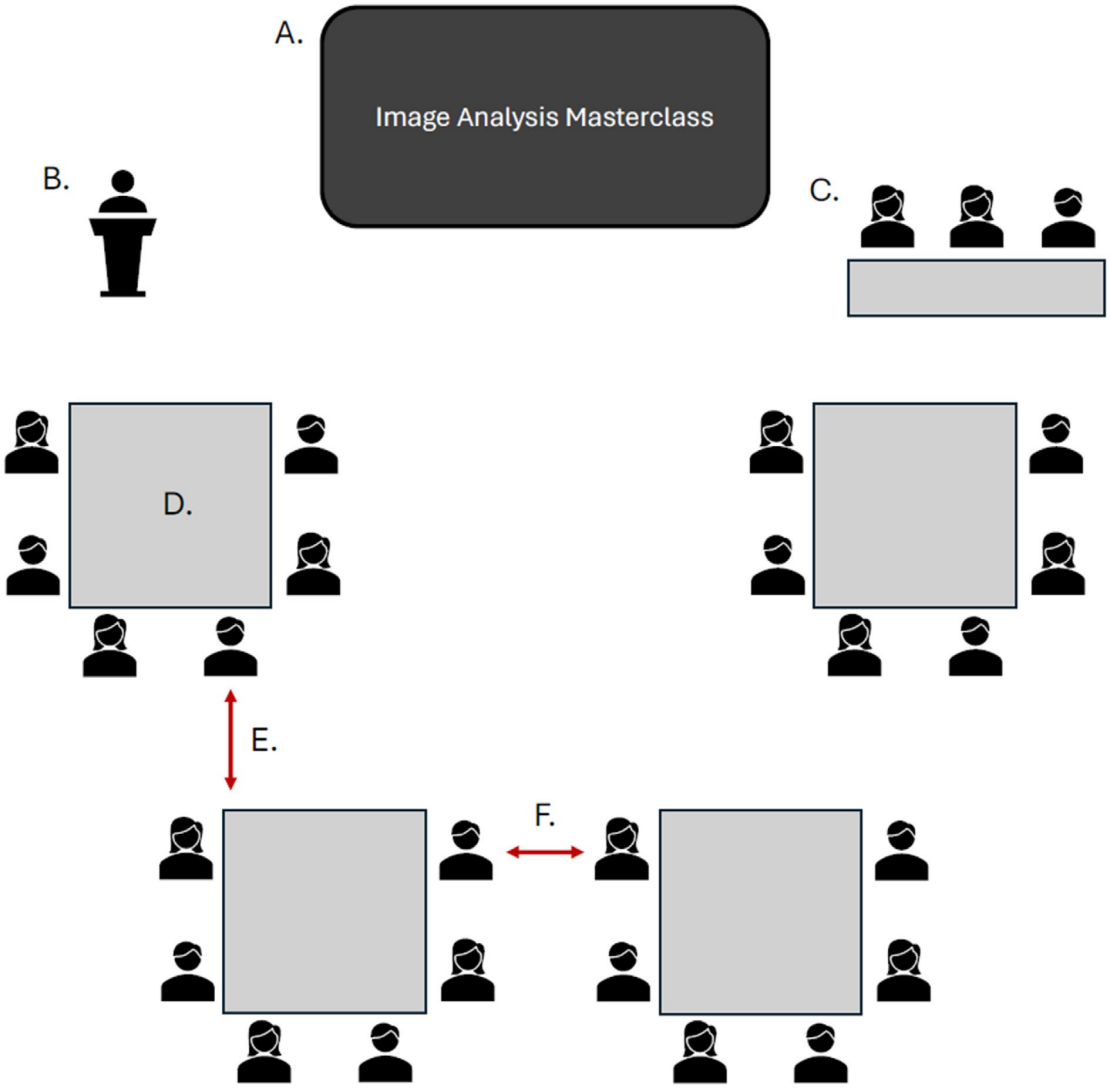
Ensure your room layout is appropriate for your chosen workshop format. Here we suggest things to consider for a group‐based workshop. (A) Ensure the screen is large enough to be clearly seen from the back of the room—this is especially important for coding sessions. (B) Ensure there is a lectern (ideally with a microphone) or table for the lead instructor for each session to stand behind. (C) If space permits, a separate table for instructors to “rest” should be provided. (D) Each table should allow enough space for each participant to comfortably use a laptop and mouse. Seats should be arranged in a manner that ensures everyone has as clear a view of the screen as possible. A minimum of one power outlet for every two participants should be available at each table. (E, F) The minimum clearance between (and around) each group should be large enough to permit instructors to pass comfortably.

Encouraging group work can be challenging if the training room is not suitable (Figure 2). Securing an appropriate room at an academic institution, especially during term time, can be difficult, so it is important to start planning early and book well in advance. For a group‐based workshop, you ideally need a meeting room with configurable chairs and tables, ample power outlets (as many rooms have an inadequate supply), fast Wi‐Fi, and a large screen or multiple smaller screens spread around the room.

Be realistic about the room's capacity. While it might be tempting to pack in as many participants as possible, ensure there is sufficient space for instructors to move around easily. Additionally, consider using a microphone for the speaker. This not only makes it easier for participants to hear but also saves the speaker's energy over the course of a long workshop.

### 2.7 What Is Your Budget—Are You Applying for Funding?

Your budget is another critical factor that will influence the workshop format. While it is possible to obtain funding for scientific workshops from sources such as The Company of Biologists (n.d.), participants are often willing to pay an attendance fee to cover costs. However, it is important to ensure that the fee is not prohibitively high, which could exclude less well‐funded researchers. Consider offering fee waivers on a case‐by‐case basis to maintain inclusivity. Additionally, information on travel grants should be circulated to support participants who need financial assistance.

### 2.8 What Do You Hope to Cover?

After deciding on the key logistics, it is time to focus on the workshop content. Your target audience will influence the broader subject matter: do you need to cover the basics, or can you assume a certain level of preexisting knowledge? You will need to decide on the balance between lectures on theory, live demonstrations, and hands‐on practicals. It is crucial to ground practical sessions with thorough theoretical explanations beforehand. This approach may cause more advanced participants to find the pace a bit slow, but it is preferable to ensure that no one is left behind due to the pace being too fast. For practical sessions, allow plenty of time for experimentation. Many participants will be attempting tasks for the first time, possibly using unfamiliar software.

**FIGURE 3:**
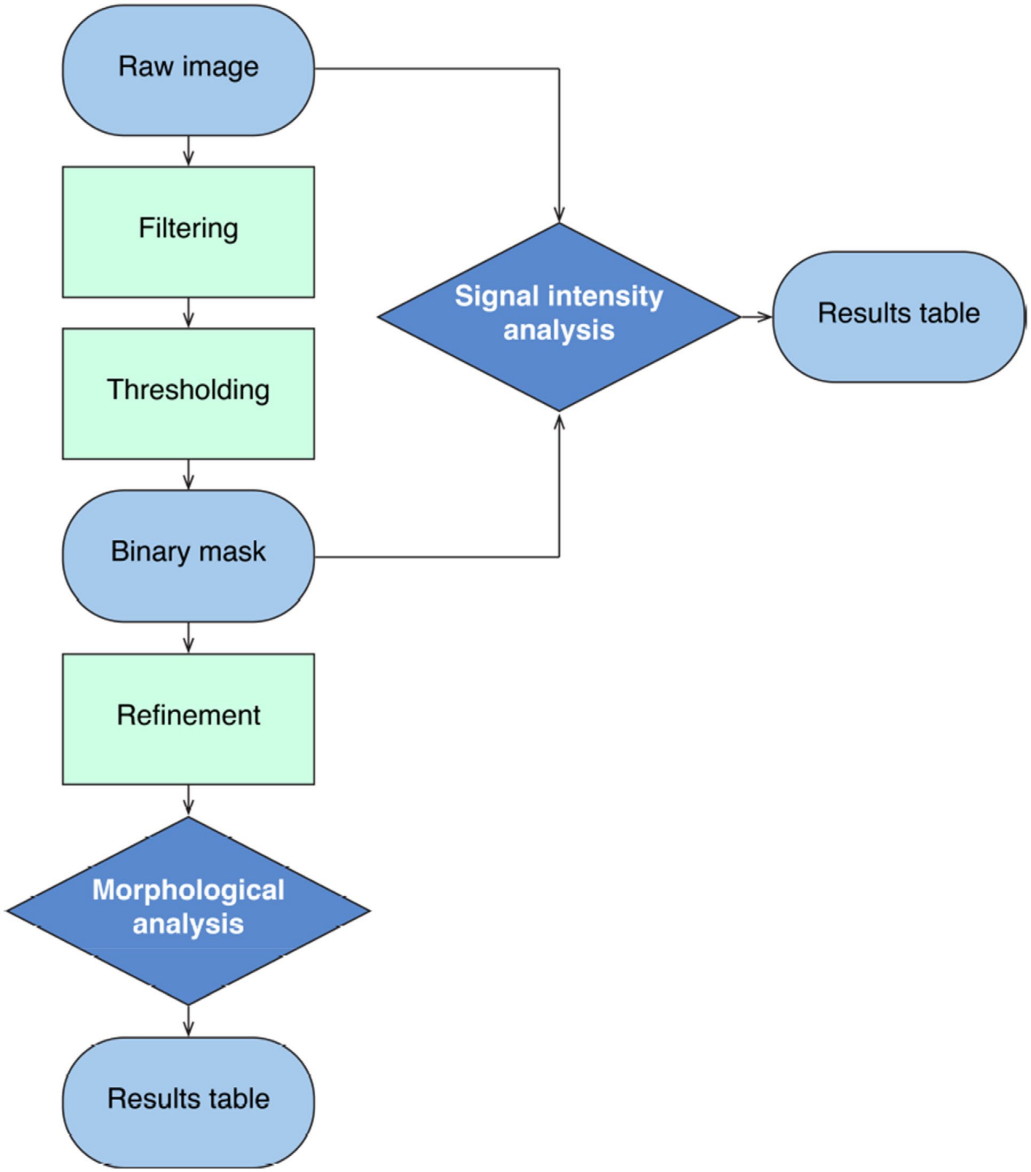
Suggested workflow for an introductory image analysis workshop. Explaining, in detail, the theory behind each workflow step, prior to any practical exercise, is important to ensure all participants fully understand why each step is necessary.

It can be tempting to cover everything, but we strongly advise against this to avoid overwhelming your participants (and avoid wasting precious time!). Focus on a specific workflow (see Figure 3) and provide all necessary background information for your participants to understand and perform each step (Barry, Marcotti, and Jones 2024). Keeping theoretical explanations as software‐agnostic as possible will help participants transfer skills across different software platforms.

Allow ample time for questions and discussions within each session. If you finish early, use the time for informal Q&A during breaks. Flexibility in course content can be advantageous; adjust topics on the fly based on the pace of participant progress. While this can be challenging for inexperienced instructors, having a few reserve slides for potential questions is beneficial.

### 2.9 What Tools Will Be Used During the Workshop?

The choice of presentation tools and software will be heavily influenced by the content you wish to cover and your target audience. Tools such as FIJI (Schindelin et al. 2012) are excellent for teaching beginners, as they are easy to install and come with built‐in functionality necessary to get started with image analysis. For more advanced users interested in automating tasks with Python, Jupyter notebooks (Kluyver et al. 2016) are an excellent resource, as they allow for the combination of documentation and code.

However, using Python‐related tools may require allocating time in the program for setting up environments and installing necessary packages. While some participants may be able to complete this setup prior to the workshop, many will likely need assistance, so be prepared to offer support during this process. If possible, consider bundling Python packages together in a single installer using the conda constructor tool (or similar) to make this process as easy as possible for participants (docs.conda.io/projects/conda/en/latest/user‐guide).

### 2.10 Will They Use Their Own Laptops?

Finally, consider whether participants will use dedicated workstations, prebuilt virtual machines (VMs) or bring their own laptops. If a training room with workstations is not available, the decision is straightforward. However, if you do have access to such facilities, each option has its advantages. Using host institute workstations can minimize technical issues, as all necessary software can be preinstalled. Network issues are also less likely, as workstations are typically Ethernet‐connected. Additionally, you would not need to worry about power outlets for everyone's laptops.

In the case of both workstations and prebuilt VMs, the likelihood of technical problems during the workshop can be reduced substantially by ensuring that all software and environments are correctly configured prior to the workshop. This can ensure that all participants begin the workshop with exactly the same software configuration and maximizes the time for practical learning.

On the other hand, installing software can be a valuable part of the learning experience, empowering participants who might typically rely on their IT department. It also ensures that participants leave the workshop with the software installed on their own laptops, enabling them to continue using it afterward.

If participants are bringing their own laptops, the probability of encountering technical issues is high. These issues can stem from participants not reading instructions, poor Wi‐Fi connectivity, or unexpected problems. Therefore, it is crucial to allocate sufficient time in the program to address technical mishaps. This time should be proportional to the complexity of the installations and inversely proportional to the reliability of the software. Hope for the best but prepare for the worst.

### 2.11 Publicizing Your Workshop

Ensuring your target audience hears about your event is key to its success, so begin publicizing as soon as possible. How you do so will depend to some extent on your intended audience—strategies for promoting a local event are likely to differ from those used to raise awareness of an international event. Nevertheless, social media posts are likely to be beneficial, regardless of the audience. Well‐designed, eye‐catching posters can also be effective. Online resources such as MicroscopyDB (microscopydb.io) should also be exploited, particularly for international workshops. It is also important not to neglect more conventional approaches, such as direct mailing (via mailing lists or otherwise), including a link to the workshop in your email signature, “plugging” the workshop during presentations (ask colleagues to do the same if possible) or good old‐fashioned word of mouth—drop it into conversations with anyone who you think might be interested.

### 2.12 Selecting Applicants

#### 2.12.1 Establishing Selection Criteria

Rather than allowing anyone to sign up on a first‐come, first‐served basis, we recommend screening applicants through an application process. There are two main reasons for this. First, there is typically a high demand for image analysis training, particularly in the life science community, often exceeding the capacity of the workshop. Second, to maximize the value of the training, it is desirable to select applicants who are going to put into practice what they learn as soon as possible. Otherwise, there is a risk that the knowledge they have gained during the workshop will be lost, resulting in wasted time on the part of both the participant and the trainer.

#### 2.12.2 Prerequisites

Using an application form to screen candidates is effective (see Box 3). The questions on the form should reflect your target audience and the aims of the workshop. In our experience, asking applicants to provide a brief statement (less than 200 words) explaining why they should be selected can be very telling. Additionally, asking about their experience with specific software packages (e.g., ImageJ/FIJI) or coding can provide valuable insights into their suitability for the workshop.

BOX 3 **Suggested questions for an introductory image analysis workshop application form.**

**Table:** 

**What is your job title?** PhD Student PostDoc Staff Scientist Group/Core Facility Lead Other	**What kind of microscopy do you routinely work with?** Bright Field Contrast Enhancing Fluorescence Super Resolution Scanning/Transmission Electron Atomic Force
**What field do you work in?** Physical Sciences Life Sciences Other	**Do you have experience with Python?** ^a^ Yes, I am very experienced. Yes, a little. No, but I have coded in other languages (e.g., MATLAB, R, IJ Macro). I have no coding experience.
**How often do you use the following software (never/sometimes/often)?** See Box 1 for examples.	**What institute are you based at?** ^b^

^a^ In our experience, people tend to overestimate their coding expertise, so be prepared for “expert” coders to make basic mistakes.

^b^ This can ensure there are not many participants from the same institution, so information can be disseminated as widely as possible.

#### 2.12.3 Minimizing Bias

By using any kind of selection process, there is of course the risk of introducing bias, conscious or otherwise. We therefore suggest anonymizing applications as far as is possible and utilizing some kind of objective scoring mechanism for the applicants. While scoring statements made by the applicants may well be somewhat subjective, other criteria (coding ability, experience with image analysis software, experience with microscopy) are easier to score and this should be taken into consideration when drafting the application form.

## 3 During the Workshop

### 3.1 Dealing With Technical Issues

Before the workshop, it is advisable to send all applicants details on any data they need to download or software they need to install, especially if they are using their own laptops. Requesting that they verify the installations were successful is also recommended. However, even with well‐prepared participants, technical issues are likely to arise, particularly with personal laptops.

The impact of these issues will depend on their nature. If the problem is widespread, pause the teaching to address the issue as a class before moving on. If the issue is specific to an individual, have one instructor attempt to resolve the problem while the rest of the class continues.

### 3.2 Managing Expectations

It is crucial to manage expectations both at the outset and in advance of the workshop. While many participants will have realistic expectations about what can be achieved in a single workshop, some may anticipate becoming fully‐fledged image analysts by the end. Address this misconception early and often. Emphasize during your introductory slides exactly what you hope to achieve during the workshop to temper expectations.

Do not hesitate to reiterate that “image analysis is hard.” If it could be mastered in a workshop lasting only a few hours or days, the demand for such workshops would be significantly lower.

**Expectation Drift**

In our experience, even with well‐defined goals and the best efforts to maintain realistic expectations, there will still be participants who will expect solutions for their specific problems. So be prepared for requests to view participants' own data and offer advice on analyzing it during the workshop. How you deal with such requests will likely depend on their complexity and whether participants have been requested to bring their own data. But be advised that agreeing to such requests can lead to follow‐up requests for additional support. Consider directing such individuals to the Scientific Community Image Forum (Rueden et al. 2019), a centralized resource that connects developers and users of open‐source image analysis tools from around the world (forum.image.sc).

### 3.3 Promoting Interaction

The more engaged the participants are, the more they will learn. Therefore, promoting interaction both with and among participants is highly beneficial. Simple methods, such as pausing to ask questions during lecture sessions or setting group tasks, can foster engagement. These group tasks can range from interspersing presentation slides with simple questions and quizzes to be discussed and answered by each group (tools such as Mentimeter, for example, can be useful for such tasks—www.mentimeter.com), to more complex “pipeline‐building” challenges, in which groups have to work together to “put into practice” what they have learned during the workshop so far. Adding a competitive element, such as awarding points to teams for correct answers, can also boost motivation. Encouraging participants to interact within their groups also helps ensure that no individual is “left behind” and everyone understands the material.

### 3.4 Ensuring Nobody Gets Left Behind

During the workshop, it is important to gauge everyone's progress as much as possible. Rather than simply setting a task and asking everyone to indicate when they are done, ask participants to provide specific solutions to particular questions. This approach helps identify which groups are grasping the material and which may be struggling. If a group consistently fails to answer questions correctly, consider diverting instructors to provide additional assistance.

The priority should be to ensure that everyone (or at least every group) achieves some predefined minimum milestone. This could be the completion of a particular challenge, such as assembling a simple pipeline or correctly executing a script. This not only provides a measurable outcome for your training but also gives participants a tangible takeaway and a sense of achievement.

It is also important to be mindful of the fact that, for a variety of reasons, different participants will learn at different rates. The most obvious cause of this, considering the global nature of science, is a language barrier. Assuming your workshop will be conducted in English (which the vast majority of international conferences and workshops are), non‐native English speakers may need additional time to assimilate information (Lenharo 2023). Therefore, it is imperative that you allow plenty of time for questions and discussion during your presentations and demonstrations to clarify points, or just to repeat yourself if something is not completely clear.

## 4 After the Workshop

### 4.1 Receiving Feedback

It is imperative to collect feedback from participants as soon as possible after the workshop, ideally before they leave the room. Positive feedback can help secure support for future workshops, while negative feedback—or better, constructive criticism—provides valuable insights into areas that require improvement. Consider allowing participants to submit feedback anonymously. While this does not allow you to follow up with them to discuss any points raised, it may make them more comfortable in providing constructive criticism.

We would also suggest that your feedback form consist of only multiple‐choice questions, as far as is possible (see Box 4 for some suggestions). While one or two optional free‐form responses are worth including for additional feedback, the less effort participants must expend to complete the form, the more responses you are likely to receive.

BOX 4 **The feedback you request may depend on the nature of your workshop. However, the following are likely to be useful in many circumstances.**

**Table:** 

**What is your job title?** This allows a more granular analysis of the feedback responses—early career researchers might appreciate certain aspects of the workshop more than senior participants (or vice versa).	**What was your overall level of satisfaction with the workshop?** Always a good idea to gauge overall sentiment, but low ratings are difficult to interpret in the absence of additional questions.
**How difficult did you find the exercises?** Probably something you will get a sense for during the workshop, but always a good idea to determine if participants found exercises too easy/difficult.	**Did you think the length of the workshop was appropriate?** This is particularly useful when you begin teaching for the first time and may find it difficult to judge how much material can be adequately covered in a given timeframe.
**Did you get enough support from the instructors and have enough opportunities to ask questions?** You may have felt that you had an adequate number of instructors, but participants may have felt differently.	**Did you find it useful to work in a group with other participants?** While we advocate a group‐based learning approach, there may be specific circumstances when this is not entirely appropriate.
**How did you find the balance between demonstrations and working on exercises?** Also very useful when beginning teaching for the first time—a theory‐heavy workshop may result in less engagement from your participants.	**Did you find the training room and associated facilities appropriate?** It's always a good idea to check these things with participants before commencing the workshop. (Can everyone see the screen? Can everyone access a power outlet? Is anyone too hot/cold?), but no harm to check afterwards to see if anything was overlooked that may have presented difficulties for participants.
**If there's anything you'd like to add, please do so here—your feedback will help us to improve future workshops.** While it's always a good idea to use multiple‐choice questions so far as possible, free‐form responses can provide some valuable insights.

It is important to note that cultural differences can significantly influence how feedback is provided. Some participants may be more reserved or indirect in their feedback due to cultural norms, while others may be more forthcoming. Being aware of these cultural differences can help you better interpret the feedback you receive and manage expectations accordingly.

### 4.2 Encouraging Further Learning

Empowering participants to continue learning beyond the workshop is essential. A good starting point is to introduce them to relevant publications that delve deeper into aspects of introductory image analysis than those covered in the workshop (Culley et al. 2024; Senft et al. 2023). Additionally, there are excellent blogs that participants may find valuable (FocalPlane n.d.; Blog n.d.). Encourage them to register on the Scientific Community Image Forum (Rueden et al. 2019), a centralized resource that connects developers and users of open‐source image analysis tools from around the world (forum.image.sc).

In addition to the many freely available tools they can access (Box 1), make them aware of key symposia and workshops in the image analysis field, such as I2K (From Images to Knowledge). Also, highlight important community initiatives, such as GloBIAS (www.globias.org) and the Royal Microscopical Society's Data Analysis in Imaging Section (www.rms.org.uk/community/science‐sections/image‐analysis.html), which provide valuable resources and opportunities for continued learning and networking.

**Interpreting Feedback**

One of the most common features of feedback we receive for our workshops is that people complain the workshop was a little too short. This could be interpreted as us attempting to squeeze too much content into too short a time period. However, given that the feedback in general is overwhelmingly positive, we prefer to interpret this as people are finding the workshops very useful and enjoyable and do not want them to end!

### 4.3 Anchoring Workshops in the Broader Academic Landscape

While our use cases below highlight successful workshops with support from prominent organizations, it is crucial to consider the broader academic landscape and the position of trainers in less privileged or less visible places. Recognizing and acknowledging trainers for their efforts, providing tangible outcomes for attendees such as attendance receipts or certificates, and implementing micro‐credentials can significantly enhance the impact and inclusivity of workshops. This can help embed workshops more effectively in postgraduate teaching and support a more inclusive academic environment.

### 4.4 Publishing Content

It is highly recommended to make all workshop content publicly available via platforms like GitHub, Zenodo, or similar resources. Additionally, we strongly suggest licensing your material appropriately, such as using the Creative Commons Attribution 4.0 International Public License (Creative Commons n.d.). This approach allows participants to revisit the material in the future and enables them to reuse your material for their own workshops, should they feel empowered to teach one.

As far as possible, teaching materials should meet the FAIR principles for scientific data management and stewardship (Wilkinson et al. 2016). That is, they should be Findable, Accessible, Interoperable, and Reusable. The following is a suggested set of minimum requirements. Publication on Zenodo ensures that a digital object identifier (DOI) will be assigned, a globally unique and persistent identifier (findable), easily accessible via any browser (accessible). All data in your teaching materials should be in commonly used formats (interoperable) and everything should be appropriately documented to ensure the material can be reused by others (reusable).

### 4.5 Share Your Experiences

Finally, consider sharing your experiences of running a workshop so others can learn from them. This can be as simple as sharing content on social media during and after the workshop, which also serves to promote your work and can be another useful avenue for receiving feedback. Additionally, consider writing a blog post for FocalPlane (n.d.) or a similar platform to share your insights. Each workshop is unique, and even seasoned trainers can benefit from learning how others conduct their sessions. You could also encourage a subset of your participants to write about their experiences, either individually or as a combined blog post.

## 5 Case Study

### 5.1 Overview

In this perspective, we reflect on our experience in teaching introductory image analysis to research and technical staff. We discuss what we find works well and where we feel there might be room for improvement, based on the participants' feedback and on our own perspective. Additionally, we discuss teaching content and learning outcomes, and we share the material used for teaching. In particular, we refer to two workshops taught in the spring of 2024, in London and Dublin, supported by the Company of Biologists and the Royal Microscopical Society. The sessions were structured as two full days, covering some image analysis foundational concepts, and the construction of the same image analysis pipeline in three different open‐source software (Fiji, Python using JupyterLab, and napari) to analyze publicly available imaging datasets. While these workshops were mostly aimed at life scientists, we believe a similar format should also work for participants with physical and material sciences backgrounds, if adapting the images used to build the workflows.

### 5.2 Participants and Logistics

The workshops were designed for 30 participants, with three instructors and one to three helpers. The participants were selected via an application form, which required them to describe their current role, availability of images to analyze, previous experience in image analysis and coding, and to write a short statement justifying their reasons for attending. Due to a high number of applications compared to the available seats, priority was given to microscopy core facility staff and more senior researchers (postdocs and principal investigators), with the idea that they could then act as trainers for their users and participants. Running this type of workshop regularly and recruiting more instructors will allow us in the future to widen the participant selection to include more PhD students.

Participants were asked to bring and use their own laptops. This choice over a computer room or remote machines was made to encourage and facilitate participants to continue their image analysis endeavors following the workshop, having already locally installed software and working examples. Participants were provided with instructions to install some software (Fiji and miniconda) about a week before the sessions and offered support for troubleshooting by emailing the instructors if needed. The choice of using miniconda over Anaconda as a Python environment manager was made to keep installation lighter. If any participant was already using Anaconda on their machine, we only asked them to update it to a recent version as the installation of miniconda and Anaconda in parallel on the same machine is not recommended. The update ensured that libmamba was used as the default solver, which is required for the successful installation of napari.

We grouped participants in teams of five, ensuring that each table had a mix of skill levels in image analysis and coding. We tried to encourage interaction and group discussions by providing exercises throughout the sessions that required one answer per group via a Google Form. Notably, the room chosen for the workshop should provide sufficient space for instructors and helpers to “roam around” the tables and offer support and feedback when required. Moreover, it should have a suitable number of power outlets to allow participants to charge their machines and standard visual and mobility accessibility requirements should be accommodated. Finally, if possible, it would be helpful to arrange dedicated access to the network with local IT departments, as we found that download speed was a limitation when many participants were simultaneously retrieving files or packages from the internet.

The costs to host such workshops can be kept relatively low and include charges for room bookings and catering, and travel/accommodation costs for trainers and helpers. It should be noted that finding suitable rooms has often proved challenging during term times, and that sufficient time for planning and bookings should be accounted for.

### 5.3 Content

The workshop content focuses on open‐source software and publicly available data, and we provide materials openly, in an effort to respect the FAIR (findable‐accessible‐interoperable‐reusable) principles. Sessions are structured in an interactive live‐coding format, encouraging participants to follow along as the instructors demonstrate concepts and build content in a step‐by‐step manner. The pace is designed to allow time for questions and discussions, and participants are requested to provide regular feedback. Colored post‐it notes are also provided to each participant following the Carpentries recommendations (The Carpentries n.d.): they can use them on the side of their screen when a task has been completed successfully (e.g., green note) or if they need support (e.g., red/pink note) so that instructors can slow down as needed while helpers solve the upcoming issues.

We opted for starting the first day with a brief introduction to foundational image analysis concepts (image formation, thresholding, segmentation, filtering), because, from our experience, users often have not been exposed to the theoretical background behind some of the basic operations they might already be performing on their images. Additionally, we covered some general content on where to find help (e.g., image.sc), and on further available resources.

Building on this foundational knowledge, we then used Fiji to build a pipeline for segmentation, counting, and morphological analysis of cell nuclei using the graphical user interface (GUI). The idea behind starting from the Fiji GUI is that this software is still considered the gold standard for image analysis in the life sciences community, as it was developed exactly for this purpose, and it is actively maintained. Moreover, it is an intuitive and valuable tool for teaching image analysis to participants and facility users, with a relatively gentle learning curve and a wide range of support material available online. The concept of automation and batch processing is then introduced to expand the analysis performed on a single image on a larger dataset, with the use of the Macro Recorder tool and ImageJ macro language.

The first session of the second day covers Python installations, as we found this to be a bottleneck for the following part, especially due to the differences between operating systems and institutional IT management on the participants' laptops. In particular, we introduce Python environments and managers and create a fresh environment via the terminal. We then installed in the newly created environment the relevant packages needed to build in JupyterLab and napari the same pipeline constructed the previous day in Fiji. Care should be taken by the trainers at this stage to make sure instructions are up to date, as installation recommendations for napari are updated regularly to accommodate the actively developed nature of the software.

We then move on to JupyterLab, using precompiled notebooks that include short exercises to allow participants to familiarize themselves with the tool, despite possibly having little or no coding experience in Python. The first notebook covers some foundational concepts on variables, operations, and arrays. The following notebooks build the same image analysis pipeline of Day 1, first on a single image, and then as a batch process on a small dataset. We decided to introduce Python as an alternative to Fiji, as it allows for more flexibility when it comes to integrating other tools (e.g., machine/deep learning) and it is more powerful and flexible for downstream data analysis, thanks to dedicated libraries developed for applications wider than bioimage analysis (e.g., scikit‐image, pandas).

Building on this introduction to image analysis in Python, we then introduce napari, and specifically the devbio‐napari distribution (github.com/haesleinhuepf/devbio‐napari). The choice of using this “battery‐included” version developed by Robert Haase provides the participants with many plugins already installed, making the building of the image analysis pipeline more streamlined. We conclude the session by demonstrating how napari can be used as a viewer from Jupyter notebooks, providing a valuable tool for 3D visualization of complex datasets.

A dedicated website was designed for each course, including the workshop schedule, the sign‐up and installation instructions, the link to the materials for download, and the relevant contact details. The website was linked to a public GitHub repo, and the slides and material were made available on Zenodo (Barry, Marcotti, and Jones 2024).

### 5.4 Feedback and Considerations

**FIGURE 4:**
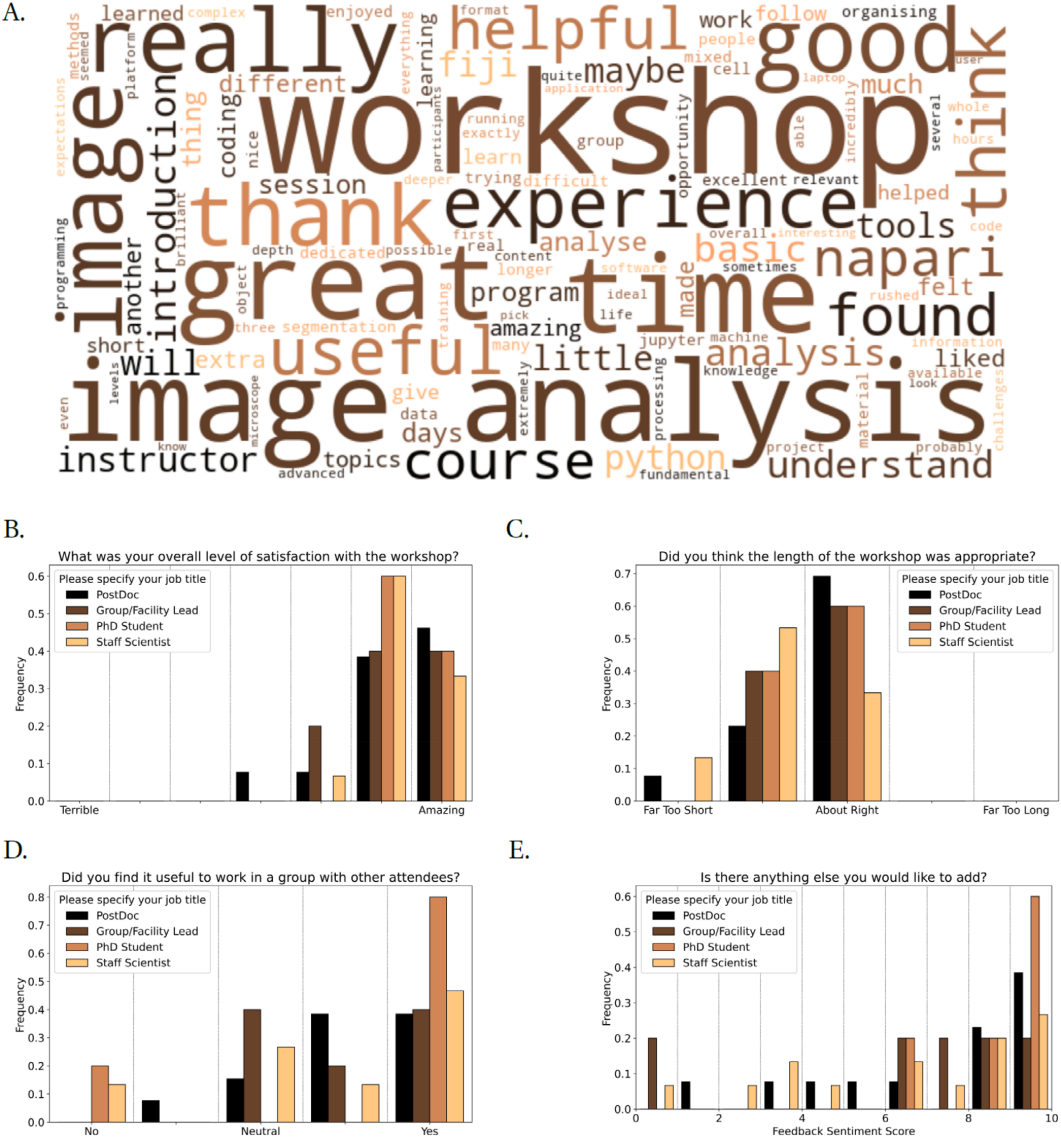
Feedback to workshops was overwhelmingly positive. (A) Wordcloud constructed from free‐form feedback responses. (B–D) Participants responses to indicated questions on the feedback form. Participants were placed into the most appropriate of four job titles. (E) Sentiment analysis of free‐form feedback responses generated using Python's NLTK module's Sentiment Analyser (Bird, Loper, and Klein 2009).

The overall feedback from participants to these workshops has been overwhelmingly positive (Figure 4). The most recurring comment is related to extending the duration of the courses to explore more advanced materials, or to allow for independent work on the topics covered. While we think there might be scope for more intermediate training, we also believe that there is a limit on what can be taught and learned with workshops of this format. The idea is to give participants a solid foundation that allows them to deepen their knowledge independently, based on their needs, and to provide them with further resources to get started in this journey.

It is often the case that participants in this type of course have a wide range of backgrounds, both in terms of theoretical and technical skills. While partially this can be addressed with an application‐based selection, some participants will still find the pace too slow or too fast for their needs. Possibly, this problem could be mitigated in the future if more introductory image analysis courses were run on a regular basis, and more local support provided by dedicated image analysts within institutions.

While many inevitable technical issues occur during the courses, participants report little concern over this, if support is provided in a timely manner so that they can follow the subsequent topics as the content builds up. This highlights the importance of the role of the helpers, as the instructor leading the session is often unable to troubleshoot all the issues in “real time.” Offering the role of helpers to junior image analysts could also provide them with more confidence in taking up instructing in future iterations of the workshops, widening the pool of available trainers.

These workshops provide an invaluable opportunity for networking, both for instructors and participants alike. (Bio)image analysts are part of a young community, whose role is yet to be fully recognized: occasions for interaction and discussion such as these are important to widen institutional support and often provide the basis for fruitful collaborations.

## 6 Conclusion

Running any training workshop is something of an art and there is certainly no one correct way to do it. The advice provided above is not intended to be prescriptive but merely reflects our experience of what has worked well when teaching image analysis. We hope the reader finds some useful tips in what we have written to incorporate into their teaching and training. But by far the most effective way to learn about the best way for you to run a workshop is to actually run one. Rest assured, you will make mistakes and things will go wrong (and your participants will forgive you for this—nothing ever goes 100% smoothly), but, with experience, you will learn how to deal with such things more and more gracefully. So, embrace the chaos and start teaching!

## Author Contributions

**Stefania Marcotti:** writing – original draft, software, data curation, methodology, conceptualization, validation, visualization. **Martin L. Jones:** writing – review and editing, software, methodology, conceptualization, validation. **Thomas J. A. Slater:** writing – review and editing. **David J. Barry:** writing – original draft, data curation, methodology, conceptualization, formal analysis, software, validation, visualization.

## Data Availability

The data that support the findings of this study are openly available in Introduction to Image Analysis at https://github.com/RMS‐DAIM/introduction‐to‐image‐analysis.

## References

[jemt24769-bib-0001] Ahlers, J. , D. A. Moré , O. Amsalem , et al. 2023. “napari: A Multi‐Dimensional Image Viewer for Python.” 10.5281/zenodo.8115575.

[jemt24769-bib-0002] Bankhead, P. , M. B. Loughrey , J. A. Fernández , et al. 2017. “QuPath: Open Source Software for Digital Pathology Image Analysis.” Scientific Reports 7: 16878. 29203879 10.1038/s41598-017-17204-5 PMC5715110

[jemt24769-bib-0003] Barry, D. J. , S. Marcotti , and M. Jones . 2024. “RMS‐DAIM/Introduction‐To‐Image‐Analysis.” 10.5281/zenodo.11072733.

[jemt24769-bib-0004] Berg, S. , D. Kutra , T. Kroeger , et al. 2019. “ilastik: Interactive Machine Learning for (Bio)image Analysis.” Nature Methods 16: 1226–1232. 31570887 10.1038/s41592-019-0582-9

[jemt24769-bib-0005] Bird, S. , E. Loper , and E. Klein . 2009. Natural Language Processing With Python. Germany, Europe: O'Reilly Media Inc.

[jemt24769-bib-0006] CC BY 4.0 Deed|Attribution 4.0 International|Creative Commons . n.d. December 10, 2024. https://creativecommons.org/licenses/by/4.0.

[jemt24769-bib-0007] Chaumont, F. d. , S. Dallongeville , N. Chenouard , et al. 2012. “Icy: An Open Bioimage Informatics Platform for Extended Reproducible Research.” Nature Methods 9: 690–696. 22743774 10.1038/nmeth.2075

[jemt24769-bib-0008] Collaborative Learning—Center for Teaching Innovation . n.d. December 10, 2024. https://teaching.cornell.edu/teaching‐resources/active‐collaborative‐learning/collaborative‐learning.

[jemt24769-bib-0009] Culley, S. , A. C. Caballero , J. J. Burden , and V. Uhlmann . 2024. “Made to Measure: An Introduction to Quantifying Microscopy Data in the Life Sciences.” Journal of Microscopy 295: 61–82. 37269048 10.1111/jmi.13208

[jemt24769-bib-0010] Deslauriers, L. , L. S. McCarty , K. Miller , K. Callaghan , and G. Kestin . 2019. “Measuring Actual Learning Versus Feeling of Learning in Response to Being Actively Engaged in the Classroom.” Proceedings of the National Academy of Sciences 116: 19251–19257. 10.1073/pnas.1821936116 PMC6765278 31484770

[jemt24769-bib-0011] FocalPlane—Where Biology Meets Microscopy . n.d. December 10, 2024. https://focalplane.biologists.com/.

[jemt24769-bib-0012] Kluyver, T. , B. Ragan‐Kelley , F. Pérez , et al. 2016. “Jupyter Notebooks—A Publishing Format for Reproducible Computational Workflows.” In Positioning and Power in Academic Publishing: Players, Agents and Agendas, 87–90. Netherlands: IOS Press.

[jemt24769-bib-0013] Laal, M. , and S. M. Ghodsi . 2012. “Benefits of Collaborative Learning.” Procedia—Social and Behavioral Sciences 31: 486–490.

[jemt24769-bib-0014] Lenharo, M. 2023. “The True Cost of science's Language Barrier for Non‐native English Speakers.” Nature 619: 678–679. 37464000 10.1038/d41586-023-02320-2

[jemt24769-bib-0015] Measure Everything…Ask Questions Later—Blog of the Carpenter‐Singh and Cimini Labs . n.d. December 10, 2024. https://carpenter‐singh‐lab.broadinstitute.org/blog.

[jemt24769-bib-0016] Rueden, C. T. , J. Ackerman , E. T. Arena , et al. 2019. “Scientific Community Image Forum: A Discussion Forum for Scientific Image Software.” PLoS Biology 17: 1–3. 10.1371/journal.pbio.3000340 PMC6602289 31216269

[jemt24769-bib-0017] Schindelin, J. , I. Arganda‐Carreras , E. Frise , et al. 2012. “Fiji: An Open‐Source Platform for Biological‐Image Analysis.” Nature Methods 9: 676–682. 22743772 10.1038/nmeth.2019 PMC3855844

[jemt24769-bib-0018] Schneider, C. A. , W. S. Rasband , and K. W. Eliceiri . 2012. “NIH Image to ImageJ: 25 Years of Image Analysis.” Nature Methods 9: 671–675. 22930834 10.1038/nmeth.2089 PMC5554542

[jemt24769-bib-0019] Scientific Meeting Grants—The Company of Biologists . n.d. December 10, 2024. https://www.biologists.com/grants/scientific‐meeting‐grants/.

[jemt24769-bib-0020] Senft, R. A. , B. Diaz‐Rohrer , P. Colarusso , et al. 2023. “A Biologist's Guide to Planning and Performing Quantitative Bioimaging Experiments.” PLoS Biology 21: e3002167. 37368874 10.1371/journal.pbio.3002167 PMC10298797

[jemt24769-bib-0021] Stirling, D. R. , M. J. Swain‐Bowden , A. M. Lucas , A. E. Carpenter , B. A. Cimini , and A. Goodman . 2021. “CellProfiler 4: Improvements in Speed, Utility and Usability.” BMC Bioinformatics 22: 433. 34507520 10.1186/s12859-021-04344-9 PMC8431850

[jemt24769-bib-0022] The Carpentries—Workshop Needs . n.d. December 10, 2024. https://docs.carpentries.org/resources/workshops.

[jemt24769-bib-0023] Wilkinson, M. , M. Dumontier , I. Aalbersberg , et al. 2016. “The FAIR Guiding Principles for Scientific Data Management and Stewardship.” Scientific Data 3: 160018. 26978244 10.1038/sdata.2016.18 PMC4792175

